# “Recent Advances in Cancer Research” Bern, November 4-5, 2022

**DOI:** 10.1038/s41420-022-01249-1

**Published:** 2022-12-14

**Sponsorship:** Publication of this supplement was funded by Nanotech Innovations LTD, who collected and prepared the abstracts for publication.

All content was reviewed and approved by the Scientific Committee at Kazan Federal University, which held full responsibility for the abstract selections.

Webpage for the event: http://cancerproteostasis.com

## The role of autophagy in Hashimoto’s thyroiditis

### Anastasia Burtseva, Zinaida Abramova

#### Laboratory of Immunopathology, Kazan Federal University, Kazan, Russia; *ORCID: 0000-0003-3749-3411*

**Introduction:** Hashimoto’s thyroiditis (HT) is currently the most common autoimmune disease in humans, and the most common cause of hypothyroidism (occurring in approximately 5% of the population). Autophagy is a multifactorial regulated cell death mechanism that may play a dual role in different thyroid diseases or at different stages of the same disease. The recent studies have confirmed that activation of both autophagy itself and various disorders in the autophagic pathway can mediate inflammatory responses and immune hyperactivation, which is closely related to the pathogenesis of autoimmune diseases, including HT.

This study has shown that the mTOR signaling pathway in T cells is closely associated with autophagy abnormalities and may play an important role in the pathogenesis of HT.

**Materials and methods:** To evaluate autophagic activity in isolated peripheral T lymphocytes, we examined these cells for the presence of autophagosomes by transmission microscopy, flow cytometry, and Western blot for autophagosomal markers. Peripheral blood mononuclear cells (PBMCs) were isolated by Ficoll-Hypaque density-gradient centrifugation. CD3^+^T cells were isolated using negative selection (DynaBeads Untouched T-cell Isolation Kit, Invitrogen), with routine purity >95%. The HT group consisted of 20 HT patients. Clinical diagnosis of HT was based on elevated serum levels of thyroid antibodies (TPOAb> 200 IU/ml). The healthy control (HC) group consisted of 29 participants.

**Results:** Autophagic vacuoles quantified by electron microscopy are also found to be significantly more frequent in T cells from HT patients compared with healthy controls. This elevated number of autophagic structures is not distributed homogeneously and appears to be more pronounced in certain T cells. Also, lipofuscin granules and multilayered autophagosomes were found in T lymphocytes of HT patients. Levels of negative regulator of autophagy, the mammalian target of rapamycin, mTOR in patients with HT is significantly higher than in T cells of healthy control group. Compared with the group of healthy donors, the levels of both autophagy initiator proteins (ATG14 and Vps34), and elongation proteins (LC3-I and LC3-II) are also higher in T cells of patients with HT. The analysis showed a correlation between the levels of LC3-I and LC3-II proteins and the functional state of the thyroid gland.

**Conclusion:** We demonstrated that mTOR activity is increased in T-cell from HT patients. These results suggest that abnormally activated mTOR activity could may be responsible for impaired autophagy, regulate the survival of autoreactive T cell during HT, and could thus lead to design new therapeutic options for treatment Hashimoto’s thyroiditis.

**Funding:** This study was part of the Kazan Federal University Strategic Academic Leadership Program (PRIORITY-2030).

**Disclosure:** The authors declare no competing interests.

## Evaluation of immune cell cytotoxic activity after interaction with tumour-derived membrane vesicles

### Ivan Filin, Kristina Kitaeva, Anna Gorodilova, Yuri Mayasin, Chulpan Kharisova, Daria Chulpanova, Valeria Solovyeva, Albert Rizvanov

#### Institute of Fundamental Medicine and Biology, Kazan Federal University, Kazan, Russia; *ORCID: 0000-0002-3661-0527*

**Introduction:** Cytochalasin B-induced membrane vesicles (CIMVs) are membrane-bound structures of various sizes, that contain proteins, lipids, nuclear and mitochondrial components. CIMVs are able to fuse with recipient cells via endocytosis. Therefore, CIMVs from tumor cells can be used to present antigens to cells of the immune system.

**Materials and methods:** CIMVs were obtained from human melanoma M-14 cells using cytochalasin B. Peripheral blood monocytes (PBMCs) were isolated by Ficoll gradient centrifugation (1,077 g/cm^3^). Differentiation of dendritic cells (DCs) from PBMCs was reached by cultivation of PBMCs with a cocktail of cytokines for 7 days. Co-cultivation of DCs and CIMVs was carried out for 48 h, and then PBMCs were added. Afterward PBMCs were co-cultivated with M-14 cells for 24 h and then M-14 cells were stained with the antibodies containing a fluorescent label for annexin V. The results were analyzed by flow cytometry.

**Results:** We analyzed the cytotoxicity activity of activated PBMCs after interaction with mature DCs using confocal microscopy. It was shown that the number of apoptosis M-14 cells increased in 20% after cultivation with activated PBMCs than after cultivation with non-activated PBMCs, and in 23% compared to control M-14 cells.

**Conclusions:** Thus, due to the ability of CIMVs to present tumor antigens to DCs and activate the antitumour immune response, CIMVs of tumor cells are a promising object for the development of therapeutic antitumour vaccines. However, further studies are needed in this area to study possible ways of modulating the immune response.

**Funding:** This study was part of the Kazan Federal University Strategic Academic Leadership Program (PRIORITY-2030) and funded by the Russian Science Foundation grant 21-74-10021.

**Disclosure:** The authors declare no competing interests.

## Functional characterization of the interaction between TG2 with p53 tumor suppressor protein in lung adenocarcinoma cells

### Yuliya Gnennaya^1^, Evgenii Smirnov^1^, Nikolai Barlev^1,2^

#### ^1^Laboratory of Regulation of Gene Expression, Saint-Petersburg, Russia; *ORCID: 0000-0002-3571-333X*

**Introduction:** Transglutaminase type 2 (TG2) is a multifunctional enzyme that mediates a wide range of covalent modifications of proteins. TG2 is involved in pathogenesis of a number of neoplastic and fibroproliferative diseases, including malignant tumors and fibrosis of the lungs, kidneys, and heart. High levels of TG2 in cells of several types of lung cancer and RCC (renal cell carcinoma) correlate with poor patient survival and increased resistance to chemotherapy drugs. Apart from the fact that TG2 inhibition suppresses the RCC tumor growth by p53-mediated apoptosis, little detail is known about the role of TG2 in modulating p53 activity in the context of lung cancer.

**Materials and methods:** We created a number of isogenic adenocarcinoma cell lines with different p53 status (wt vs mut) and explored the potential mechanism of TG2-mediated effects on the intracellular distribution of p53 and subsequently, its functions. We validated the ability of these proteins to interact using GST pull-down and CoIP assay. Furthermore, WB and qPCR, to analyze the alterations in the levels of p53-dependent proteins. To evaluate the effect of TG2 on p53-mediated sensitization and cell death of the obtained cell lines upon treatment with etoposide combined with clinically approved TG2 inhibitor cysteamine, we performed a battery of assays, including cell cycle analysis, colony formation test, xCELLigence proliferation analysis, etc.

**Results:** Together, our data strongly suggest that TG2 down-regulates the expression and activity of wild-type p53 but not its mutant form. This effect was abrogated by blocking the cross-linking activity of TG2 by cysteamine, which results in the augmented stability of the p53 protein.

**Conclusion:** Collectively, TG2 can be considered as an oncogene that inactivates p53 response to DNA damage.

**Funding:** The work was supported by the grant from RSF # 20-15-00189.

**Disclosure:** The authors declare no competing interests.

## Tamoxifen suppresses NLRP3 priming via miR-223 in breast cancer independently from ERα

### Shaimaa Hamza^1^, Ekaterina E. Garanina^1^, Svetlana F. Khaiboullina^1^, Gulcin Tezcan^2*^

#### ^1^Institute of Fundamental Medicine and Biology, Kazan Federal University, 420008 Kazan, Russia; ^2^Department of Fundamental Sciences, Faculty of Dentistry, Bursa Uludag University, Bursa 16059, Turkey; *ORCID ID: 0000-0002-5012-4760*

**Introduction:** Tamoxifen is drug used for breast cancer chemotherapy. However, the resistance to tamoxifen was demonstrated in some cases. The mechanisms of this resistance remain largely unknown, while the inflammation was suggested as playing an important role. Inflammation could be mediated by Nod-like receptor protein 3 (NLRP3) inflammasome, which was shown to promote proliferation, survival, metastasis, angiogenesis, and immunosuppression of breast cancer cells. The NLRP3 priming step could be regulated by post-transcriptional modification using microRNA (miRNA). MiR-223, a miRNA with tumor suppressing capacity, was shown to block translation of NLRP3 mRNA and suppress the breast cancer cell growth.

**The aim of the study:** In this study, NLRP3 inflammasome activation in breast cancers cell lines, MCF-7 (ER-α^+^) and, MDA-MB-231(ER-α^−^), was analyzed. Also, the effect of tamoxifen on regulation of miR-223/NLRP3 axis in these cell lines was investigated.

**Materials and methods:** Lipopolysaccharide (LPS)/ATP was used to activate NLRP3. Glybenclamide/LPS served as a control for NLRP3 suppression. Western blot was used to confirm NLRP3 activation and Gasdermin D (GSDMD) cleavage, while IL-1β secretion was shown by ELISA. Apoptosis and proliferation kinetics were analyzed by immune blotting of BAX protein, Annexin V and cell proliferation assay. Statistical analysis was done using one-way ANOVA with Tukey’s analyses, Kruskal–Wallis one-way analysis of variance, independent sample t-test and linear regression analysis.

**Results:** LPS/Tamoxifen suppressed NLRP3 transcription, induced miR-223 expression and IL-1β secretion as compared to LPS-only in both cell lines (*p* < 0.001). Additionally, NLRP3 activation reduced the tumor cell growth in both cells lines, whereas suppressing NLRP3 could result in tumor cell line specific growing pattern.

**Conclusions:** Our findings suggest that tamoxifen could suppress the expression of NLRP3 by inducing miR-223 expression in breast cancer cells. However, the tamoxifen mediated suppression of NLRP3 increased the proliferation of tumor cells lacking ER-α, whilst it attenuated proliferation of ER-α-expressing breast cancer cells.

**Funding:** This work was supported by Russian Science Foundation grant #21-74-00048.

**Disclosure:** The authors declare no competing interests.

## Mesenchymal stem cells derived microvesicles suppress activation of T and B-lymphocytes

### Sirina Kurbangaleeva^1^, Sevindzh Kletukhina^1^, Marina Gomzikova^1^

#### ^1^Laboratory of Intercellular communication, Kazan Federal University, Kazan, 420008, Russia; *ORCID: 0000-0003-1637-1888*

**Introduction:** Mesenchymal stem cells (MSCs) derived extracellular vesicles (EVs) demonstrate immunosuppressive effects on T cells, B-cells, dendritic cells, and macrophages. However, EVs is difficult to obtain in the desired amounts, so we used cytochalasin B to mass-produce microvesicles. The aim of our work was to evaluate the immunosuppressive activity of induced microvesicles (iMV) of MSCs using model of peripheral blood mononuclear cells (PBMCs) activation using phytohemagglutinin (PHA) in vitro.

**Material and methods:** Treatment of MSCs with cytochalasin B (10µg/ml) and vortexing induce iMV-MSCs formation in a large scale. PBMCs were pre-treated with iMV-MSCs for 24 h followed by incubation with PHA (10 µg/mL) for 72h. The percent of activated CD25 expressing T-helpers (CD4+CD25+), B-cells (CD19+CD25+), and T-cytotoxic lymphocytes (CD8+CD25+) in the PBMCs was analyzed by flow cytometry.

**Results:** We found that iMVs themselves do not influence on activation status of PBMCs, since no differences between expression of CD25+ in control PBMCs and iMV-MSCs treated PBMCs were detected. Treatment with PHA-induced activation in T-helpers by 34.64 times (p = 0.000005), in B-cells by 3.12 times (p = 0.0002), and in T-cytotoxic lymphocytes by 87.36 times (p = 0.000001) compared with control. Conversely, pre-treatment of PBMCs with iMV-MSCs decreased subsequent PHA-induced activation of PBMCs by 2.3 times in T-helpers (0.0006), by 2.02 times in B-cells (0.0004), and by 4.38 times in T-cytotoxic cells (0.000005).

**Conclusion:** We found that iMV-MSCs show significant suppression on phytohemagglutinin-induced activation of PBMCs in vitro.

**Funding:** This work was funded by RSF research project №21-75-10035 and supported by the Kazan Federal University Strategic Academic Leadership Program (PRIORITY-2030).

**Disclosure:** The authors declare no competing interests.

## Targeting mitochondrial functions in tumor-associated macrophages as a strategy for cancer therapy

### Adelya Mullakhmetova^1^, Yana Mukhamedshina^1^, Marina Gomzikova^1^, Albert Rizvanov^1^, Nick Barlev^1^, Nikita Markov^2^, Hans-Uwe Simon^1,2^, Anna Brichkina^3^

#### ^1^Laboratory of Molecular Immunology, Institute of Fundamental Medicine and Biology, Kazan Federal University, Kazan, Russia; ^2^Institute of Pharmacology, University of Bern, Bern, Switzerland; ^3^Center for Tumor and Immune Biology, Clinic of Gastroenterology, Endocrinology, Metabolism and Infectiology, Philipps University of Marburg, Marburg, Germany; *ORCID: 0000-0001-8707-9900*

**Introduction:** Tumor-associated macrophages (TAMs) represent a major population of immune cells infiltrating solid tumors and are often associated with a poor patient prognosis. Pro-tumorigenic M2-like macrophages tend to exert immune-suppressive effects, favouring tumor progression. Pro-tumorigenic functions of TAMs require massive metabolic rearrangements linked to mitochondria and rely on intact mitochondrial respiration. Therefore, targeting the mitochondrial metabolism appears to be a promising strategy to diminish the pro-tumorigenic effect of macrophages.

**Materials and methods:** We utilized a mouse model with conditional deletion of *Opa1* in macrophages (Opa1^∆M^) to induce mitochondrial dysfunction. We cultivated tumor cells with bone marrow-derived macrophages of wild-type or *Opa1-/-* or their conditioned media. The activation status of macrophages was evaluated by stimulation with IL4 into M1-subtype or IFNγ+LPS into M2 followed by qPCR analysis of marker genes.

**Results:** Opa1^∆M^ macrophages could not stimulate the proliferation of cancer cells. This phenotype does not depend on phagocytic abilities of macrophages, but is strongly associated with the secretome of macrophages and their failure in re-programming into the pro-tumorigenic M2-like subtype.

**Conclusions:** Opa1 regulates activation of TAMs, leading to the production of secreted factors required for tumor growth. Drugs inducing mitochondrial dysfunction in macrophages might be prominent candidates for anticancer therapy.

**Funding:** This work was supported by the grant from Russian Government No. 075-15-2021-600.

**Disclosure:** The authors declare no competing interests.

## Methyltransferase Set7/9 regulates sensitivity of HER2-positive breast cancer cells to the genotoxic stress

### Dmitry Myadelets, Sergey Parfenyev, Julia Vasileva, Oleg Semenov, Olga Fedorova, Oleg Shuvalov, Alexey Petukhov, Alexandra Daks*

#### Laboratory of Gene expression Regulation, Institute of Cytology RAS, St Petersburg, Russia; *ORCID: 0000-0003-0495-1244

**Introduction:** HER2-positive phenotype of breast cancer (BC) is considered to be one of the most aggressive form of this disease due to high intensity of tumor growth and metastasis formation. Elevated expression of HER2 is an unfavorable prognostic marker for BC patients. Additionally, it was repeatedly shown that HER2-positive BC cells are characterized by the ability to acquire the resistance to genotoxic therapy.

Set7/9 is a methyltransferase that modifies a list of histones and non-histone targets with lysine residues thus affecting their functions, stability and cellular localization. Set7/9 was shown to methylate cancer-associated transcription factors including p53, E2F1, estrogen receptor α (ERα), NFkB, STAT3, YAP, androgen receptor (AR) and others.

In this study we investigated the role of Set7/9 in genotoxic stress response and cisplatin resistance of HER2-positive human BC cells SKBR3.

**Materials and methods:** Set7/9 knockdown was performed by lentiviral transduction of BC cells with subsequent puromycin selection. The cytotoxic effect of cisplatin was investigated using MTT test and by registration of cell index via xCelligence cell analyzer. The apoptosis level was assessed using annexin V/PI double staining and flow cytometry analysis. The expression of excision repair factors was determined both by qPCR and western blot analysis.

**Results:** We revealed that knockdown of Set7/9 in SKBR3 cells led to acquisition of resistance to cisplatin and to decrease of apoptosis level under cisplatin treatment. We also demonstrated that Set7/9 suppression causes activation of such DNA excision repair factors as ATR, Fen1, Rad51, MPG, APE1, XRCC1 both at mRNA and protein levels.

**Conclusions:** According to our data, methyltransferase Set7/9 affects the sensitivity of HER2-positive BC cells to cisplatin treatment via regulation of excision repair factors under genotoxic conditions. These findings contribute to investigation of drug resistance acquisition mechanisms and allow to consider Set7/9 expression as a biomarker of genotoxic therapy response of HER2-positive BC.

**Funding:** This work was supported by the grant from RSF#19-75-10059.

**Disclosure:** The authors declare no competing interests.

## Evaluation of viability, cytokine profile changes and pluripotency genes of colorectal cancer spheroids cells after interaction with tumor and stem vesicles

### Aleksei Ponomarev^1^, Anna Kurnenkova^1^, Zarema Gilazieva^1^, Albert Rizvanov^1^, Valeriya Solovyeva^1^

#### ^1^Laboratory of Gene and cell technology, Institute of Fundamental Medicine and Biology, Kazan Federal University, Kazan, Russia; *ORCID: 0000-0003-2320-6119*

**Introduction:** Extracellular vesicles are membrane structures that have a relevant role in intercellular communication because they have the ability to transport lipids, transcription factors, mRNA, and proteins. There is evidence of a special role of vesicles in cancer progression. Therefore, the study of the vesicle effect on tumor spheroids is important. The aim of this work is to study the effect of cytochalasin B-induced membrane vesicles (CIMVs) of colorectal cancer spheroids in vitro.

**Materials and methods:** In this study, сolorectal cancer cell line (HCT-15) was used to create tumor spheroids. Glioblastoma cell line (SNB-19) and adipose derived mesenchymal stem cells (MSCs) were used to isolate MVs. Vesicles from SNB-19 (SNB-19 CIMVs) and MSCs (MSC CIMVs) were isolated using 10 μg/ml of cytochalasine B and a series of sequential centrifugations. The addition of SNB-19 CIMVs and MSC CIMVs to spheroids was carried out at concentrations of 1, 2 and 5 μg. The effect of SNB-19 CIMVs and MSC CIMVs was analyzed using flow cytometry, multiplex analysis, and real-time PCR.

**Results:** After addition of MSC CIMVs, there was no significant change in cell viability in the spheroid. However, addition of SNB-19 CIMVs to colonosphere, there was a dose-dependent increase cell viability. OCT4 and Nanog mRNA levels were increased after addition of SNB-19 CIMVs but decreased after MSC CIMVs. Cytokine analysis showed significant differences in 40 major cytokines in colonospheres with SNB-19 CIMVs and MSC CIMVs.

**Conclusions:** Thus, it was shown the possible effect of SNB-19 CIMVs and MSC CIMVs on tumor spheroids. Further research of this mechanism effects is necessary.

**Funding:** This study was part of the Kazan Federal University Strategic Academic Leadership Program (PRIORITY-2030) and funded by the Russian Science Foundation grant 21-74-10021.

**Disclosure:** The authors declare no competing interests.

## Downregulation of Set7/9 increases migration activity of cancer cells through Zeb1 expression activation

### Oleg Semenov, Sergey Parfenyev, Olga Fedorova, Oleg Shuvalov, Alexey Petukhov, Alexandra Daks*

#### Laboratory of Gene expression Regulation, Institute of Cytology RAS, St Petersburg, Russia; *ORCID: 0000-0003-0495-1244

**Introduction:** Migration ability is one of the key characteristics of cancer cells aggressiveness due to its contribution to metastasis formation. Due to epithelial-to-mesenchymal transition process (EMT) the epithelial cells attached to basement membrane acquire the ability to migrate into the blood vessels with subsequent formation of new cancer foci. Zeb1 is one of the key EMT-orchestrating transcription factors that suppresses the expression of epithelial markers such as E-cadherin, cytokeratins and integrins and contributes to stabilization of mesenchymal factors e.g. vimentin and N-cadherin. In addition to EMT Zeb1 was shown to contribute to other cancer-associated characteristics such as drug resistance, stemness and cellular senescence. Dysregulation of Zeb1 and Zeb1-controlled cellular processes are tightly associated with formation and progression of different cancer types including breast and lung cancers.

Methyltransferase Set7/9 acts as a transcription regulator due to its ability to methylate histones H3, H1, H1.4, H2A and H2B thus affecting the genes expression. Additionally, Set7/9 was shown to methylate different transcription factors e.g. p53, E2F1 and NFkB and as a consequence to affect gene expression indirectly. In this study we focused on Set7/9 effect on Zeb1 expression and the role of Set7/9 in regulation of migration potential of cancer cells.

**Materials and methods:** H1299 and A549 human lung cancer cells and MDA231 human breast cancer cell line were used in this study. The migration activity of cancer cells with different Set7/9 status was investigated using scratch test and cell index registration (xCelligence cell analyzer). The levels of Zeb1 and markers of epithelial and mesenchymal phenotypes were studied both by qPCR and western blot analysis.

**Results:** As a result of this study, we showed that Set7/9 silencing led to activation of migration potential, while Set7/9 overexpression on the contrary suppressed the motility of tested cell lines. We also demonstrated that Set7/9 regulates Zeb1 and E-cadherin expression, that elucidates the mechanism of the observed effect. Thus, we revealed the new mechanism regulating cancer cell migration ability through Set7/9-Zeb1-E-cadherin axis.

**Conclusions:** We revealed that methyltransferase Set7/9 affects the migration potential of cancer cells and Zeb1 expression. Since metastasis formation remains a scourge of oncology, this study may contribute to the development of the effective strategies of anticancer therapy.

**Funding:** This work was supported by the grant from RSF# 19-75-10059.

**Disclosure:** The authors declare no competing interests.

## Evaluation of microvesicles stability using flow cytometry approach

### Sevindzh Kletukhina^1^, Marina Gomzikova^1^

#### ^1^Laboratory of Intercellular communication, Kazan Federal University, Kazan, 420008, Russia; *ORCID: 0000-0003-1637-1888*

**Introduction:** Microvesicles (MVs) are spherical membrane structures released from the cell surface and ranging in size from 50 to 2000 nm. Today, the use of MSCs derived MVs in medicine as therapeutic agents and drug delivery vectors is of great interest. Therefore, it is important to develop high throughput methods for assessing the yield and integrity of isolated or stored MVs. The aim of our work was to develop a method for assessing the yield and integrity of MVs using the flow cytometry approach.

**Materials and methods:** MVs were obtained from MSCs using 10 µg/ml cytochalasin B, which is an agent for the cell cytoskeleton disorganization and mass production of MVs. MVs were loaded with CFDA SE dye to monitor their integrity. MVs were resuspended in saline and stored at 4°C, -20°C or at 25°C, freeze dried and stored at -20°C. Quantity and integrity of MVs were analyzed using flow cytometry with enhanced detector (BD FACS Aria III BD Bioscience, USA).

**Results:** We found that storage of MVs in saline at 25°C for 28 days led to decrease of MVs amount by 43%. Whereas the integrity of MVs was preserved at 64%. Lowering the storage temperature to 4°C allows to extend the shelf life of MVs in 4 times to 112 days with the same MVs parameters. Next, we evaluated the stability of MVs suspended in saline and stored at –20°C. After 112 days of storage, the total number of MVs decreased by 20.5%, while the integrity was preserved at 71%. Widely used method for long-term storage of bioactive drugs is freeze-drying. We found that freeze-drying and storage of MVs during 112 days at –20°C led to reduction of MVs amount by 51%, with the percent integrity of the MVs being estimated at 86%.

**Conclusion:** Thus, evaluation of MVs amount and integrity using the flow cytometry method demonstrated that freezing and storage at –20°C is the most suitable option for storing MVs in solution. This allows to maintain a high percentage of integrity and high delivery efficiency by MVs.

**Funding:** This work was funded by RSF research project №21-75-10035 and supported by the Kazan Federal University Strategic Academic Leadership Program (PRIORITY-2030).

**Disclosure:** The authors declare no competing interests.

## Interleukin-8 overexpression in tumor modulates progression in colorectal cancer

### Irina Bogomolova^1,2^, Dinara Dolgova^1^, Inna Antoneeva^1,3^, Ilseya Myagdieva^1^, Tatyana Abakumova^1^, Tatyana Gening^1^

#### ^1^Ulyanovsk State University, Ulyanovsk, Russia; ^2^*Federal Scientific and Clinical Center for Medical Radiology and Oncology Federal Medical and Biological Agency of Russia, Dimitrovgrad*, Russia; ^3^Regional Clinical Oncologic Center, Ulyanovsk, Russia; *ORCID: 0000-0003-3331-8632*

**Introduction:** Currently, a search is underway for specific molecular markers for predicting the sensitivity of colon tumors to chemotherapy. Interleukin-8 (CXCL8) is considered as a promising pro-inflammatory marker of carcinogenesis associated with tumor angiogenesis, invasion through activation of the epithelial–mesenchymal transition, secondary neutrophil chemotaxis into the tumor zone. The aim of this work is to evaluate the level of CXCL8 in the tumor as a marker of progression in colorectal cancer (CRC).

**Materials and methods:** We isolated RNA using Sileks-MagNa magnetic particles (Sileks, Russia) in 49 FFPE tumor samples from patients with stage II-III CRC. Next, we performed quantitative RT-PCR using Sybr Green dye. When calculating the normalized expression of CXCL8 (ΔΔСq), the GAPDH gene was used as a referee gene. The Kaplan-Meier test (STATISTICA 13.0 (StatSoft, USA) was used to assess the effect of the level of CXCL8 in the tumor on progression-free time.

**Results:** We have found that the expression of CXCL8 in cells of adenocarcinoma of the intestine with low differentiation (Med 8.770 (1.127–15.114) is statistically significantly higher than in the group with moderate and high differentiation (p1 = 0.004; p2 = 0.012). When comparing groups with different responses to chemotherapy, it was found that in the group of patients with disease progression (against the background of chemotherapy), the level of CXCL8 mRNA in the tumor was statistically significantly higher compared to patients with early relapse and without relapse for more than 3 years (p1 = 0.0008; p2 = 0.001).

Thus, an increase in CXCL8 expression in the primary tumor increases the relative risk of CRC progression after standard chemotherapy. Dependence on the age of patients, the stage of the disease, the presence of mutations in the EGFR-signaling pathway on the level of CXCL8 in the tumor was not revealed. Probably, the effect of CXCL8 on progression is mediated through modulation of the inflammatory response in the microenvironment, activation of tumor stem cells.

**Conclusions:** Tumor overexpression of CXCL8 is associated with short progression-free time in CRC patients after standard FOLFOX/HELOX therapy and can be considered as an independent marker of progression.

**Disclosure:** The authors declare no competing interests.

## Characterization of microglia activation in a model of spinal cord injury different severity in vitro

### Elvira Akhmetzyanova^1^, Anna Timofeeva^1^, Yana Mukhamedshina^1^

#### ^1^Kazan Federal University, Kazan, Russia; *ORCID:0000-0002-5751-6759*

**Introduction:** Inflammatory reactions that occur after spinal cord injury (SCI) are a protective mechanism of the organism aimed at minimizing the consequences of injury. However, these mechanisms do not always aim to the preservation of the neural tissue functionality. It is considered that the acquisition of a certain phenotype by microglia/macrophages is one of the key processes that determine the outcome of post-traumatic reactions in the spinal cord. Here we studied microglia activation and M1/M1 polarization in a model of mild, moderate and severe SCI in vitro.

**Materials and methods:** SCI different [mild (1.5 m/s, n = 20), moderate (2.5 m/s, n = 20) and severe (4 m/s, n = 20)] severity was induced by an impactor (Impact One ™ Stereotaxic Impactor, Leica) at Th8 level. After 3, 7, 14, and 60 days post injury (dpi) experimental and intact rats were anesthetized with overdosed isoflurane anesthesia and intramuscular injection of Zoletil (20mg/kg, Virbac Sante Animale). Then a fragment of the spinal cord obtained from the site of injury/Th8 was prepared and homogenized. To modeling the SCI in vitro, spinal cord extracts were added at 4-5 days cultivation of microglia isolated from neonatal rat cerebral cortex. To analyze cells by flow cytometry, we used markers for CD86, CD45, and CD206. To characterize the cytokine profile of microglia supernatants cultivated under different conditions, multiplex analysis was performed using the Bio-Plex Pro Rat Cytokine 23-Plex Immunoassay (Bio-Rad).

**Results:** It was shown, microglia have a consistently high level of CD86 expression in the first week of cultivation. The expression level of CD45 under different conditions of microglia cultivation ranged from 40 to 60%. The expression of CD206 had the lowest values in a model of mild and moderate SCI at 14 and 60 dpi. According to the results of multiplex analysis, significant changes in the concentration of TNF-α, IFN-γ and MCP-1 between the studied groups were found. It is noteworthy that the expression of all studied cytokines was consistently high when microglia were cultivated with an intact spinal cord.

**Conclusion:** It was shown that the severity of SCI has a decisive influence on microglia activation and M1/M2 polarization proceeds equally actively in all models of SCI in vitro. Multiplex analysis and flow cytometry data demonstrated activation of both M1 and M2 microglia in models of mild, moderate and severe SCI.

**Funding:** This study was supported by the Russian Foundation for Basic Research (to YM) (Grant No. 20-34-90060) and was part of the Kazan Federal University Strategic Academic Leadership Program (PRIORITY-2030).

**Disclosure:** The authors declare no competing interests.

## Recognition of cysteine mutant forms of the sodium-dependent phosphate transporter NaPi2b by monoclonal antibodies in ovarian cancer cells OVCAR-8

### Leisan Bulatova^1^, Daria Savenkova^1^, Daria Reshetnikova^1^, Arina Timonina^1^, Alsina Nurgalieva^1^, Vera Skripova^1^, Mikhail Bogdanov^2^, Ramziya Kiyamova^1^

#### ^1^Research laboratory Biomarker, Institute of Fundamental Medicine and Biology, Kazan Federal University, Kazan, Russia; ^2^McGovern Medical School at The University of Texas Health Science Center at Houston, Houston, USA; *ORCID: 0000-0001-6696-8477*

**Introduction:** The sodium-dependent phosphate transporter NaPi2b is a target for monoclonal antibody (mAb) therapy since it’s overexpressed in several malignancies. The extracellular domain (ECD) of the NaPi2b (188-361 a.a.) contains 4 cysteines and the MX35 epitope (324-338 a.a.). It is known that DTT reduces disulfide bonds, limiting MX35 recognition by mAbs in Western blot (WB) analysis. We suggest that cysteines around the MX35 epitope form disulfide bonds, altering the NaPi2b ECD conformation.

**Materials and methods:** NaPi2b mutants with cysteine-to-alanine substitutions at positions 303, 322, 328 and 350 were generated. The mutant forms were transfected into OVCAR-8 ovarian cancer cells and analyzed with mAbs using Western blot and flow cytometry.

**Results:** OVCAR-8 cells expressing wild-type NaPi2b showed a specific signal only without DTT as usual, while all NaPi2b cysteine mutants showed no signal in WB even without DTT. According to the results of flow cytometry, the proportion of MX35-positive OVCAR-8 cells that produce cysteine mutants of NaPi2b is significantly lower compared to the wild type of the transporter.

**Conclusions:** Cysteines C303, C322, C328, and C350 are implicated in the creation of disulfide bonds within ECD of NaPi2b. This is an essential step in the production of the NaPi2b ECD conformation, which is necessary for the recognition of the MX35 epitope by mAbs.

**Funding:** This paper has been supported by the Kazan Federal University Strategic Academic Leadership Program (PRIORITY-2030) and partly supported by by Russian Science Foundation Grant RCF 20-14-00166.

**Disclosure:** The authors declare no competing interests.

## Application of multigene panels in hereditary cancer predisposition testing

### Sergey Nikolaev^1^, Anastasia Danishevich^1^, Airat Bilyalov^1,2^, Natalia Bodunova^1^, Igor Khatkov^1^, Oleg Gusev^2,3,4^

#### ^1^The Loginov Moscow Clinical Scientific Center, Moscow, Russia. ^2^Kazan Federal University, Kazan, Russia. ^3^Graduate School of Medicine, Juntendo University, Tokyo, Japan. ^4^Endocrinology Research Centre, Moscow, Russia; *ORCID: 0000-0003-0673-3590*

**Introduction:** Approximately 5 to 10% of cancer cases are associated with hereditary cancer predisposition syndromes (HCPS). Early identification of HCPS is important for the choice of the individual monitoring and treatment strategies.

**Materials and methods:** Study cohort included 1119 probands from Russia: 1062 (94.9%) cancer patients with clinical signs of HCPS and 57 (5.1%) healthy individuals with cancer in family history. NGS analysis of 76 HCPS genes was performed using a custom Roche NimbleGen panel, which encompassed exonic and intronic regions of the genes.

**Results:** We found that 34.0% (381/1119) of the patients enrolled had pathogenic/likely pathogenic variants. 54 (27.4%) out of total 197 unique genetic alterations hadn’t been previously described in the literature, 9 (4.6%) out of these variants were located in *BRCA1/BRCA2* genes. The predominant number (59.3%) of genetic variants was identified in the *BRCA1/BRCA2* genes associated with breast and ovarian cancer syndromes. *CHEK2* was the second most commonly altered gene with the total of 29 (7.6%) identified variants: 4 likely pathogenic and 25 pathogenic variants. 155 (40.6%) genetic variants were found in other 35 cancer-associated genes of variable penetrance: 12 (3.1%) variants in high-penetrance genes (*CDH1, MLH1, MSH2, TP53*) and 143 (37.5%) genetic alterations in the genes with mild to low penetrance. Among the interesting findings, we would like to highlight a breast cancer case with a c.-39-1_-39del variant located outside the 5’UTR region of *BRCA2* gene, which is rarely included in gene panels.

**Conclusion:** Multigene panel testing allows for a differential diagnosis and identification of a high-risk group for oncological diseases. The inclusion of non-coding gene regions into HCPS gene panels is highly important for identification of rare spliceogenic variants with high penetrance.

**Disclosure:** The authors declare no competing interests.

## Ce^3+^,Tb^3+^:YF_3_ nanoparticle-polymer -“radaclorin” conjugates for X-ray induced photodynamic therapy

### Alexey S. Nizamutdinov, Elena V. Lukinova^1^, Nail Shamsutdinov^2^, Pavel V. Zelenikhin^2^, Alina Khusainova^1^, Maxim S. Pudovkin^1^

#### ^1^Institute of physics, Kazan Federal University, Kazan, Russia, ^2^Institute of Fundamental Medicine and Biology, Kazan Federal University, Kazan, Russia; *ORCID: 0000-0001-8707-9900*

**Introduction:** Photodynamic therapy (PDT) is a modern and non-invasive form of therapy, used in the treatment of cancers and non-oncological diseases. It is based on the use of photosensitizers, which are accumulated in pathological tissues. The modern photosensitizers are activated by visible light which cannot penetrate the biological tissues deeper than 1 cm. Hence, the conventional PDT can be used only for superficial diseases. There is an approach allowing using X-ray irradiation in order to overcome these limitations by creating conjugates of nanoparticles and photosensitizers. Thus, the main objectives of the study were to create the Ce^3+^,Tb^3+^:YF_3_ nanoparticles-“radaclorin” conjugates and to study biological activity (cytotoxicity and cellular uptake).

**Materials and methods:** Ce^3+^,Tb^3+^:YF_3_ nanoparticles were synthesized via co-precipitation method. The A549 (human lung carcinoma) cells were purchased in Russian collection of vertebrate cell cultures, Russian Academy of Sciences, St. Petersburg, Russia. We utilized transmission electron microscopy method in order to visualize the cell uptake process. PVP and PEI polymers were used for conjugation procedure. The UV laser was used as a substitution of X-ray irradiation.

**Results:** The efficiency of the conjugation was controlled by shortening the lifetime curves of the doping ions. The efficient radii between doping ions and radaclorin molecules were in the 5.0 – 7.1 nm range. The conjugates are non-toxic in micromolar concentrations toward A549 cells. Both PVP – and PIE-based conjugated are effectively uptaken by A549 cells via micropinocytosis. The conjugated are packed into 200 – 1500 nm vesicles. According to the flow cytometry measurements, the intensity of side scattered light (SSC) of PIE-based conjugates in two times bigger compared to PVP-based one. It reveals, that the PIE-based conjugates are uptaken more effectively.

**Conclusions:** Taking into consideration the fact the PVP-based conjugates are non-stable and they form precipitate on the bottom of the beaker, the Ce^3+^,Tb^3+^:YF_3_ nanoparticle-PVP -“radaclorin” conjugates are more effective for X-ray induced photodynamic therapy.

**Funding:** This work was carried out in accordance with the Strategic Academic Leadership Program "Priority 2030" of the Kazan Federal University of the Government of the Russian Federation.

**Disclosure:** The authors declare no competing interests.

## Efficacy of drugs targeting mitochondria in inhibiting the proliferation of colorectal cancer cells

### Sirina Kurbangaleeva^1^, Gulnaz Sharapova^1^, Nikita Markov^2^, Marina Gomzikova^1^, Anna Brichkina^3^, Albert Rizvanov^1^, Hans-Uwe Simon^1,2^

#### ^1^Laboratory of Molecular Immunology, Kazan Federal University, Kazan, Russia; ^2^Institute of Pharmacology, University of Bern, Bern, Switzerland; ^3^Center for Tumor and Immune Biology, Philipps University of Marburg, Marburg, Germany; *ORCID: 0000-0002-9404-7736*

**Introduction:** Mitochondrial aberrations are often found in tumor cells of different origin, including colorectal cancer (CRC). Mitochondrial dysfunctions are usually associated with the inability of the electron transport chain (ETC) to sustain high levels of oxidative phosphorylation due to the impaired activity of ETC complexes. Moreover, impaired ETC often leads to severe adjustments within the TCA cycle leading to the accumulation of fumarate. In this work, we explored how pharmacological induction of mitochondrial dysfunction and the subsequent switch to glycolysis, induced by treatment of cells with the ETC complex I and III inhibitors actinomycin A and rotenone, respectively, affects the survival and proliferation of CRC cells. In addition, we evaluated the effect of increased concentrations of fumarate by treating CRC cells with the cell permeable derivate dimethyl fumarate.

**Materials and methods**. Crystal violet staining of viable cells was implemented and their proliferation rates were measured using CellTrace Violet assay. Additional experiments were performed in starvation conditions (2% FBS) or during the growth of the cells in spheroids. Drugs were tested in the concentration range of 156 nM – 2 μM on four different CRC cell lines: SW480, SW620, SW48 and HCT-15 during a time period of 0-96 hours. CRC cell lines were chosen based on different bioenergetic axes and characterized by different levels of mitochondrial activity.

**Results:** Drugs targeting mitochondrial complexes of the ETC limited proliferation of CRC cell lines within both nanomolar and micromolar ranges of concentrations. Moreover, the effect of the decreased proliferation of cancer cells was observed in both glycolytic cells (HCT-15) and cells relying on mitochondrial respiration (SW480), suggesting that cancer cells are sensitive to the ETC targeting independently of their metabolic programs. On the other hand, treatment with dimethyl fumarate did not affect the proliferation of CRC cell lines (SW620, SW48, HCT-15). Interestingly, dimethyl fumarate at nanomolar concentrations promoted a minor increase in proliferation of SW480 cells, suggesting a possible modulating effect of fumarate on the cell cycle. In addition, the observed effect was confirmed in the settings of spheroid growth and starvation (2% FBS), suggesting that dimethyl fumarate indeed upregulates proliferation of SW480 cells.

**Conclusion:** Targeting of mitochondrial ETC complexes appears to be an effective strategy to limit the expansion and growth of CRC cells independently of their metabolic profiles. In contrast, dimethyl fumarate either does not affect the proliferation rates of CRC cells or promotes a minor increase in proliferation of some cancer cell lines like SW480.

**Funding:** This work was supported by the grant from Russian Government #075-15-2021-600.

**Disclosure:** The authors declare no competing interests.

## Bioenergetic heterogeneity of commonly used glioma cell lines

### Gulnaz Sharapova^1^, Sirina Kurbangaleeva^1^, Nikita Markov^2^, Marina Gomzikova^1^, Anna Brichkina^3^, Albert Rizvanov, Hans-Uwe Simon^1,2^

#### ^1^Laboratory of Molecular Immunology, Kazan Federal University, Kazan, Russia; ^2^Institute of Pharmacology, University of Bern, Bern, Switzerland; ^3^Center for Tumor and Immune Biology, Philipps University of Marburg, Marburg, Germany; *ORCID: 0000-0002-9404-7736*

**Introduction:** Gliomas are brain tumors originating from glial cells and are characterized by poor prognosis and very limited treatment options. Developing of new therapeutic options to treat gliomas depends on the understanding of their molecular properties and features.Currently, numerous drugs targeting different cellular metabolic and bioenergetic axes of cancer cells are undergoing clinical trials. However, the efficiency of these drugs is highly dependent on the bioenergetic profiles and dependencies of examined cancer cells. In this study, we investigated the metabolic profiles of commonly used glioma cell lines to select for potential therapeutic targets. Moreover, our study proposes a new level of characterization of glioma cells separating them by their bioenergetic capacity.

**Materials and methods**. In order to obtain bioenergetic profiles of six glioblastoma (A172, LN428, D247MG, LN18, LN229, T98G) and two astrocytoma (U251, LN319) cell lines, we utilized the Seahorse XFe96 analyzer. The assay was performed upon subsequent injections of oligomycin, FCCP, rotenone/actinomycin A and 2-DG. This design of assay allowed us to calculate 9 parameters related to glycolysis and mitochondrial respiration. To minimize the interference of external factors, the metabolic profiles of all 8 cell lines were measured simultaneously. The number of seeded cells varied in the range of 40 to 60 thousand owing to their difference in size. The final results were normalized using the initial seeding coefficients. In order to avoid additional variability induced by an uneven proliferation rate of examined cell lines, the cells were seeded on poly-L-lysine coated plates allowing for rapid adhesion and immediate execution of the assay.

**Results:** Bioenergetic profiling of examined cells separated them into three distinct clusters. Within the first cluster, the cell lines A172, LN319, LN428, T98G were characterized by both high levels of glycolysis and mitochondrial respiration. In contrast, cell lines D247MG, LN18, LN229 were less metabolically active and displayed attenuated levels of glycolysis and mitochondrial activity. Interestingly, the highest level of glycolysis and relatively low mitochondrial respiration was detected in U251 cell line suggesting the presence of mitochondrial dysfunction in these cells. Further studies are required to understand how bioenergetic clustering fits current molecular and WHO-based classifications of glioma and correlates with such parameters as survival, progression of the disease and sensitivity to treatments.

**Conclusion:** Simultaneous measurement of bioenergetic profiles is a powerful tool allowing to investigate the heterogeneity of cancer cells and cluster them by their metabolic activity. Commonly used glioma cell lines are characterized by distinct bioenergetic programs.

**Funding:** This work was supported by the grant from Russian Government #075-15-2021-600.

**Disclosure:** The authors declare no competing interests.

## Inhibition of TGF-β receptors of bladder cancer cells affects their mechanical properties as measured by nanopipette–based scanning ion-conductance microscopy

### Anastasiia Sapach^1,2^, Roman A. Akasov^2^, Nikita Savin^3^, Petr Gorelkin^3^, Gleb B Sukhorukov^1,4,5^, Andrei V Zvyagin^2^

#### ^1^Skolkovo Institute of Science and Technology, Moscow, Russia; ^2^Sechenov First State Medical University, Moscow, Russia; ^3^The National University of Science and Technology MISIS, Moscow, Russia; ^4^Siberian State Medical University, Tomsk, Russia; ^5^Queen Mary University of London, London E1 4NS, United Kingdom; *ORCID: 0000-0002-5745-2177*

**Introduction:** Epithelial–mesenchymal transition (EMT) is a fundamental process that governs dissemination of single carcinoma cells from the primary tumors. Remodeling of the actin cytoskeleton as well as modulation of extracellular matrix occurs within EMT and is associated with cell stiffness. Cell stiffness is usually measured by atomic force microscopy that generally needs cell fixation and labor-intensive sample preparation. The aim of this research was to study mechanical properties of live bladder cancer cells when blocking EMT by inhibition of TGF-β receptors by using nanopipette–based scanning ion-conductance microscopy (SICM).

**Materials and methods:** T24 cell line established from a human urinary bladder cancer patient was employed in the experiment. 5 × 10^5^ cells were treated with 10 µM of inhibitors (A-83-01 or A-77-01) and 5 ng/ml of TGF-β in 24 h, and, following washes, were scanned at room temperature. Cell topology and stiffness maps were acquired using SICM (ICAPPIC ltd, UK) by sampling cells as reported elsewhere. A nanopipette were used as the probe pulled from borosilicate blanks, outer/inner diameters, 1.2 mm/0.69 mm, respectively, to produce nanopipettes with the radius ranged from 40 to 50 nm. Topography and measurements of the mechanical properties were recorded in “hopping mode” over areas of 40 × 40 µm with the resolution 0.3 µm. Setpoint for topography was 0.5% and for the stiffness mapping 1 % and 2 %. Image processing was performed using the «SICMImageViewer» software.

**Results:** Stiffness measurement of single cell mechanical properties via Scanning Ion-conductance Microscopy is a novel method, which enables simultaneous topography mapping and stiffness mapping. Cell stiffness of TGF-β-induced/inhibition-blocked EMT of bladder cancer cells was measured using SICM. Control cell stiffness measurement showed 1.7±0.2 kPa, when TGF-β treated cells decreased value (0.3±0.05 kPa). Cells incubated with 10 µM of inhibitors (A-83-01 or A-77-01) and 5 ng/ml of TGF-β were significantly (p<0.05) stiffer (0.7±0.1 kPa) than cells incubated with 5 ng/ml of TGF-β only. The cell height exhibited similar trend, i.e., cells incubated with 5 ng/ml TGF-β featured higher profile than these of negative control or TGF-β/A-83-01 treated cells. Cells incubated with 10 µM of inhibitors (A-83-01 or A-77-01) maintained their membrane stiffness level even in the presence of 5 ng/ml of TGF-β.

**Conclusions:** SCIM was successfully employed as a method for studying mechanical properties of living T24 cells with activation/ inhibition of EMT.

**Funding:** The work was funded by the Siberian State Medical University’s development program Priority 2030 and by the Ministry of Science and Higher Education of the Russian Federation, the contract 075-15-2021-709, RF-2296.61321X0037.

**Disclosure:** The authors declare no competing interests.

## UCNP-based Photoluminescent Nanomedicines for Theranostics of HER2-positive tumors

### Evgenii L. Guryev^1^, Daria K Bausheva^1^, Natalia Y Shilyagina^1^, Irina V. Balalaeva I^1^, Sergey M. Deyev S.M^2,3^, Andrei V Zvyagin^1,3,4^

#### ^1^Lobachevsky State University of Nizhny Novgorod, Nizhny Novgorod, Russia; ^2^Shemyakin-Ovchinnikov Institute of Bioorganic Chemistry, Russian Academy of Sciences, Moscow, Russia; ^3^Institute of Molecular Theranostics, Sechenov University, Moscow, Russia; ^4^MQ Photonics Centre, Macquarie University, Sydney, Australia; *ORCID: 0000-0001-8799-2257*

**Introduction:** Development of new approaches for diagnosis and therapy of tumors (taken together, termed theranostics) - one of the most dynamic areas of the life sciences, where new nanomaterials afford new opportunities. In virtue of their unique optical properties, upconversion nanoparticles (UCNP) have shown promise for cell and live animal imaging. A combination of agents, a bacterial exotoxin PE40 genetically fused with a targeting protein DARPin (targeted toxin DARPin-LoPE) and radioactive beta-emitters ^90^Y were assembled into an UCNP nanocomplex that was characterized and deployed in cancer treatment experiments to exhibit a profound super-additive therapeutic effect in cells and live animal models. The binding specificity of as-produced theranostic nanomedicine and its toxic effect on HER2-positive tumor cells were studied and reported.

**Materials and methods:** UCNPs of the structure NaYF_4_:Yb:Tm were coated with a two-layer shell of organic polymers. The targeted toxin DARPin-LoPE was attached to polymer-coated UCNPs by chemical conjugation. The developed conjugation protocol enabled achievement of the desired orientation of the shell molecules and protein modules and preserve their functional activity. The specificity of the interaction of UCNP complexes with SKOVip-kat human ovarian adenocarcinoma cells was studied using fluorescence microscopy, while the cytotoxicity was assessed by an MTT method.

**Results:** The mean hydrodynamic diameter and zeta potential of as-produced UCNP-DARPin-LoPE complexes were measured to be 234 ± 29 nm and -54 ± 9 mV, respectively, indicating colloidal stability. The UCNP-DARPin-LoPE complexes were designed to target HER2-positive tumor cells due to specific interaction with target molecules on their surface. The role of these molecules is the HER2 tumor marker receptor. DARPin protein exhibited high affinity to the epidermal growth factor receptor and served as the targeting agent to HER2-expressing cells. To demonstrate the selective accumulation of UCNP-DARPin-LoPE-s on the HER2-expressing cell surface, we employed fluorescence microscopy tuned to excite and acquire UCNP photoluminescence signal, where SKOVip-kat cells transfected to express far-red fluorescence proteins were used as the HER2-positive tumor cell model. No UCNP cell binding was detected in negative controls, including polymer-coated UCNPs with no targeting protein decoration and HER2-negative cells. The cytotoxicity study showed a pronounced combined effect of the ^90^Y beta-emitters and the DARPin-LoPE toxins on SKOVip-kat cells overexpressing the HER2 receptor. Preliminary in vivo experiments showed selective accumulation of UCNP-DARPin-LoPE-s in peritoneal tumor foci in mice formed by SKOVip-kat cells.

**Conclusions:** Theranostic UCNP-DARPin-LoPE nanocomplexes were able to selectively bind to tumor cells overexpressing the HER2 receptors owing to a DARPin targeting module. Two toxic modules, ^90^Y beta-emitters and the DARPin-LoPE-s conspired to achieve a combined therapeutic effect. The obtained results showed potential of UCNP-DARPin-LoPE nanocomplexes diagnosis and therapy of HER2-positive tumors.

**Funding:** This work was supported by Ministry of Education and Science of the Russian Federation: UNN “Priority 2030” Strategic Academic Leadership Program (Project No. H-418-99_2022-2023).

**Disclosure:** The authors declare no competing interests.

## Reactivation of tumor suppressor p53 by aminobenzothiazole derivatives

### Raniya Khadiullina^1^, Damir Davletshin^1^, Elvina Khusainova^1^, Regina Mirgayazova^1^, Vitaly Chasov^1^, Matthias Baud^2^, Emil Bulatov^1^

#### ^1^Kazan Federal University, Institute of Fundamental Medicine and Biology, Kazan, Russia. ^2^University of Southampton, Department of Chemistry, Southampton, United Kingdom; *ORCID: 0000-0003-3521-5995*

**Introduction:** The *TP53* gene is mutated in all human cancers and the p53 protein is an essential component of the cell response induced by various genotoxic stresses. The presence of mutations violates the tertiary structure of the DNA-binding domain p53, which leads to destabilization of the protein, its partial denaturation and loss of activity. A promising therapeutic strategy for treating a wide range of human malignancies involves using small molecule therapeutics to reactivate mutant p53. First, we evaluated the cytotoxic effect of aminobenzothiazole derivatives on normal human fibroblast cell line (HSF) and four human adenocarcinoma cell lines with various p53 status (wild type: A549; mutant: MIA PaCa-2 ^p53(R248W)^, OVCAR-3 ^p53(R248Q)^; and knockout: MCF-7 ^p53(−/−)^). After that we identified gene expression alterations in human p53 signaling pathway induced by these compounds.

**Materials and methods:** Cells were treated with compounds at a concentration range of 2.5-120 μM or 1% DMSO (vehicle control) and assessed after 24 h and 48 h using colorimetric MTS assay. Quantitative RT-RCR and western blotting were performed according to standard protocol after treatment cell lines with 60 μM compounds or vehicle control for 48 h. **Results**. MTS assay revealed a significant dose- and time-dependent cytotoxic effect of the compounds on all cell lines. However, this effect was more pronounced in cancer cells than in normal cells, and cancer cells with p53 wild-type or knockout tended to be less sensitive than those with mutant p53. Furthermore, the compounds showed upregulated p53 target gene transcription in cancer cells with mutant p53. At the same time no substantial modulating effects were observed in cell line with wild-type p53.

**Conclusions:** Our study helped to further reveal molecular mechanisms underlying the reactivation of different mutant p53 forms. This will be important for the development of novel, individually tailored anticancer drugs.

**Funding:** The study was funded by RSF grant 22-24-20034 and strategically supported by Kazan Federal University Strategic Academic Leadership Program (PRIORITY-2030).

**Disclosure:** The authors declare no competing interests.

## Effect of p53 activator Nutlin-3a on populations of immune cells obtained from healthy donor and patient with multiple sclerosis

### Irina Ganeeva, Aygul Valiullina, Ekaterina Zmievskaya, Albert Rizvanov, Emil Bulatov

#### Institute of Fundamental Medicine and Biology, Kazan Federal University, Kazan, Russia; *ORCID: 0000-0003-4041-5000*

**Introduction:** The role of p53 in autoimmune diseases has been demonstrated in many models. Multiple sclerosis (MS) is a chronic autoimmune disease that affects myelin. Currently, there are 44 new cases of MS per 100,000 population worldwide diagnosed every year, and the number of patients is constantly growing. However, the role of p53 in the regulation of MS is still unclear.

In this study we evaluated the effect of p53 activator Nutlin-3a on cytokine secretion by immune cells obtained from a healthy donor and an MS patient.

**Materials and methods**. We used peripheral blood mononuclear cells (PBMCs) of a healthy donor and an MS patient and divided them into 3 groups: untreated, treated with 10 μM and 40 μM Nutlin-3a. PBMCs were incubated with Nutlin-3a for 24 hours. To evaluate the effect of Nutlin-3a on p53 activation and expression of p53-dependent genes we assessed *p21*, *Bax* and *PUMA* expression levels by quantitative RT-PCR. In addition, flow cytometry analysis was carried out for cells stained with antibodies to CD45, CD3, CD4, CD20 and p53 protein. Cell supernatants were collected and tested by cytokine multiplex analysis using Bio-Plex 200 system (Bio-Rad, USA).

**Results and conclusion**. RT-PCR analysis demonstrated increased expression levels p53-dependent genes (*p21, Bax, PUMA*). Flow cytometry analysis revealed increased proportion of p53 positive T- and B-cells both in cells from healthy donor and MS patient after co-incubation of PBMCs with Nutlin-3a. At the same time, the ratio of the analyzed populations of immune cells did not change significantly. According to multiplex cytokine analysis, in healthy donor cells Nutlin-3a increased the level of IP-10, reduced IL-10, G-CFS, MCP-1, TNF-α, VEGF and did not affect IL-1b, IL-1ra, IL-15, INF-γ, RANTES. In MS patient cells, Nutlin-3a increased the levels of IL-1b and TNF-α, decreased IL-1ra, IL-10, G-CSF, and VEGF and did not affect IL-15, IFN-γ, IP-10, MCP-1, RANTES. Our results show that activation of p53 by Nutlin-3a affects cytokine secretion in PBMCs of both healthy donor and MS patient. This suggests a potential role of p53 protein in regulation of immune-related processes in MS.

**Funding:** This work was supported financially by the Strategic Academic Leadership Program of Kazan Federal University (PRIORITET-2030).

**Disclaimer:** The authors declare no conflict of interest.

## Pharmacological activity of invertebrate steroid hormone, ecdysterone, in human normal and cancer cells

### Oleg Shuvalov, Yuilia Kirdeeva, Elyzaveta Fefilova, Alexandra Daks, Olga Fedorova

#### Institute of Cytology RAS, St Petersburg, Russia; *ORCID:*

**Introduction:** In human, the insect hormone 20-hydroxyecdysone (ecdysterone) displays a number of beneficial pharmacological activities including antioxidant, hypoglycemic, hepato-, cardio- and neuroprotective. Ecdysterone is broadly known among athletes for its anabolic activity, due to which it is used worldwide to increase physical fitness. In addition, a number of studies has recently shown its antineoplastic activity. However, to date, there is no understanding of either the molecular mechanisms or mode of ecdysterone’s activity in different types of cells including malignant ones.

**Materials and methods:** We have carried out the comparison of ecdysterone-induced biological effects in mouse (C2C12) and rat (L8) myoblasts, mouse embryonic fibroblasts (MEFs), human fibroblasts (DF2) and non-small cell lung adenocarcinoma cell lines (H460, H1299, A549 and H1975) in a dose-dependent manner. We have studied the influence of ecdysterone on their proliferation, cell cycle, level of reactive oxygen species (ROS), membrane potential of mitochondria, energy metabolism, autophagy and the resistance to chemotherapeutic drugs.

**Results:** Ecdysterone has slightly decreased the proliferation of all cells. It significantly reduced glycolysis and respiration and induced autophagy. At the molecular level, ecdysterone strongly suppressed the expression of c-Myc and some glycolytic enzymes which are its transcriptional targets. In addition, ecdysterone notably augmented ROS production in cancer models whereas reduced it in normal cells.

**Conclusions:** Ecdysterone suppresses energy metabolism in cancer cells and induce ROS production. Due to safety and plethora of valuable pharmacological properties, ecdysterone should be in focus of researches as potential adjuvant which may help cancer patients to prevail consequences of chemotherapy as well as make it more efficient.

**Funding:** This work was supported by the RSF grant No. 21-75-10138.

**Disclosure:** The authors declare no competing interests.

## Regulation of autophagy flux by E3 ubiquitin ligase Pirh2

### Alexandra Daks^1^, Sergey Parfenyev^1^, Oleg Shuvalov^1^, Anastasia Gudovich^1^, Nick Barlev^1^, Olga Fedorova^1^

#### ^1^Institute of Cytology, Russian Academy of Sciences, 194064, St Petersburg, Russian Federation; *ORCID: 0000-0003-1382-4204*

**Introduction:** Autophagy is a catabolic process aimed at the restoration of the energy supply in cells under various forms of stress. Initially, autophagy was considered as one of the cell death mechanisms and only later it was recognized as a protective mechanism. Human Pirh2 (p53-induced RING-H2 protein is an E3 ubiquitin ligase that mediates p53 degradation. In addition to p53, Pirh2 was shown to attenuate Chk2, p73, p63, polH, c-myc, and p27 thus

regulating cell proliferation, cell cycle progression, and DNA repair.

**Materials and methods:** We cultivated tumor cells with knockdown of Pirh2 and control. The level of autophagy was evaluated by western blot analysis followed by qPCR analysis of marker genes.

**Results:** We have revealed E3 ligase, Pirh2, that affects the expression of different genes that participate in all steps of autophagy. Using Western blot and immunofluorescent analysis we demonstrated that Pirh2 positively regulates the accumulation of LC3-II in cancer cell lines.

**Conclusions:** We have uncovered a novel function of Pirh2 in the regulation of autophagy in cancer cells.

**Funding:** This work was supported by the grant from RSF No. 18-75-10076.

**Disclosure:** The authors declare no competing interests.

## Germline genetic variants of ERBB3 in triple-negative breast cancer

### Maria Zolotykh^1^, Alfya Nesterova^2^, Airat Bilyalov^1,3^, Albert Gimranov^2^, Julia Filina^1^, Eugenia Boulygina^1^, Albert Rizvanov ^1^, Regina Miftakhova^1^

#### ^1^Institute of Fundamental Medicine and Biology, Kazan Federal University, Kazan, Russian Federation, ^2^Tatarstan Regional Clinical Cancer Center, Kazan, Russian Federation, ^3^Moscow Clinical Research Center named after A.S. Loginov MHD, Moscow, Russian Federation; *ORCID: 0000-0003-2473-5514*

**Introduction:** Receptor tyrosine-protein kinase ERBB3, also known as human epidermal growth factor receptor 3 (HER3), is a gene that encodes a member of the epidermal growth factor receptor (EGFR) family. Preclinical studies demonstrated that mutations in ERBB3 gene can promote oncogenesis in a ligand-independent manner in breast cancer (ВС). Five missense mutations in ERBB3 are associated with breast neoplasms. Furthermore, HER3 overexpression is associated with poor prognosis in patients both with HER2-positive and the triple-negative breast cancer (TNBC). Germline genetic variants in ERBB3 affect response to therapy agents in HER2-positive BC patients. The aim of the study was to determine the germline genetic variants of ERBB3 gene in TNBC patients, study the correlation with overall and progression-free survival.

**Materials and methods:** The study involved 20 patients with TNBC and the control group, consisting of 21 volunteers without family history of cancer in three generations. Genomic DNA was isolated from peripheral blood mononuclear cells. Exome sequencing was performed on Illumina’s NextSeq platform using SureSelect clinical research exome V2 system (Agilent, USA). Statistical data processing was carried out using a chi-square test and Kaplan-Mayer survival analysis. Samples were collected according to KFU Ethical Committee-approved protocol.

**Results:** We have identified three SNPs rs773123, rs56017157, rs2271188 in patients with TNBC and healthy volunteers. The rate of rs773123 genetic variant was significantly higher in TNBC compared to control group (*p* = 0.007). Presence of rs773123 did not affect overall survival (p = 0,644) in our study. Missense-mutation rs773123 in ERBB3 gene leads to S1119C protein change. Several studies confirm that presence of SNP rs773123 was associated with worse relapse-free survival, decreased sensitivity to doxorubicin, carboplatin and trastuzumab in HER2-positive BC patients.

**Conclusions:** Germline single nucleotide polymorphism rs773123 in ERBB3 might be associated with increased risk of triple-negative breast cancer.

**Funding:** This work was part of Kazan Federal University Strategic Academic Leadership Program (PRIORITY-2030).

**Disclosure:** The authors declare no competing interests.

## Genetic varians of DNA repair genes in cisplatin-resistant ovarian cancer cells

### Aisylu Sagdeeva^1^, Julia Filina^1^, Yana Shamsutdinova^2^, Rezeda Galimova^2^, Alexey Sabirov^2^, Aigul Rakhmatullina^1^, Maria Zolotykh^1^, Rimma Mingaleeva^1^, Albert Rizvanov^1^, Regina Miftakhova^1^

#### ^1^Institute of Fundamental Medicine and Biology, Kazan Federal University, Kazan, Russia; ^2^Tatarstan Regional Clinical Cancer Center, Kazan, Russia; *ORCID: 0000-0002-9318-2004*

**Introduction:** High activity of the DNA repair system defined by the genetic variants may contribute to the chemotherapy resistance, which is one of the most common complications in the ovarian cancer treatment. The aim of the study was to dedermine the clinically significant single nucleotide polymorphisms involved in ovarian cancer development and chemotherapy resistance.

**Materials and methods:** FFPE surgical resections and blood samples (n = 86) of OC patients were obtained from the Tatarstan Regional Clinical Cancer Center. All patients underwent cisplatin monotherapy (n = 42) after surgery and were observed for at least 6 months after the end of chemotherapy. According to the disease-free interval patients were divided into platinum-resistant (disease progression during therapy or within 6 months after) and platinum-sensitive (patients without relapse for more than 6 months after therapy). Control blood samples (n = 37) were obtained from healthy volunteers without cancer cases in three generations. DNA was isolated from sections of the normal tissue of patients with ovarian cancer and control blood samples to assess germinal mutations. Genomic DNA sequencing was carried out using the target NGS panel covering 20 genes of the DNA repair and replication system coding regions. Statistical analysis was performed using Fisher’s exact test; p≤0.05 was considered statistically significant. Samples were collected according to KFU Ethical Committee-approved protocol.

**Results:** Sequencing analysis revealed 356 single nucleotide variants in 20 genes of the DNA repair and replication system in healthy volunteers and patients with OC. Eleven SNVs in ATM, BRCA1, FEN1, MUTYH, POLI and POLQ were associated with the ovarian cancer development. We also observed higher rate of germline APEX1 c.T444G SNV in platinum-resistant compared to platinum-sensitive OC patients: homozygous GG genotype was more frequent in the resistant patients in the comparison with TT+TG (p = 0.045). OGG1 c.C977G genotype was associated with disease-free survival in OC: median time was 0.756 months for GG and 11.441 months for CC and CG carriers (p = 0.009).

**Conclusions:** Polymorphic variants GG444 of the APEX1 gene and GG977 of the OGG1 gene may be promising biomarkers for predicting the effectiveness of therapy and ovarian cancer outcome.

**Funding:** This work was supported by the Russian Foundation for Basic Research grant 17-00-00263 and performed in accordance with the Kazan Federal University Strategic Academic Leadership Program (PRIORITY-2030).

**Disclosure:** The authors declare no competing interests.

